# No Association of Hair Zinc Concentration with Coronary Artery Disease Severity and No Relation with Acute Coronary Syndromes

**DOI:** 10.3390/biom12070862

**Published:** 2022-06-21

**Authors:** Ewelina A. Dziedzic, Jakub S. Gąsior, Agnieszka Tuzimek, Justyna Paleczny, Mirosław Kwaśny, Marek Dąbrowski, Piotr Jankowski

**Affiliations:** 1 Medical Faculty, Lazarski University in Warsaw, 02-662 Warsaw, Poland; 2 Department of Internal Medicine and Geriatric Cardiology, Centre of Postgraduate Medical Education, 01-813 Warsaw, Poland; agnieszkatuzimek@gmail.com (A.T.); piotr.jankowski@interia.pl (P.J.); 3 Department of Pediatric Cardiology and General Pediatrics, Medical University of Warsaw, 02-091 Warsaw, Poland; jgasior@wum.edu.pl; 4 Department of Pharmaceutical Microbiology and Parasitology, Faculty of Pharmacy, Wroclaw Medical University, 50-556 Wroclaw, Poland; justyna.paleczny@student.umed.wroc.pl; 5 Institute of Optoelectronics, Military University of Technology, 00-908 Warsaw, Poland; miroslaw.kwasny@wat.edu.pl; 6 Department of Cardiology, Bielanski Hospital, 01-809 Warsaw, Poland; mardab@vp.pl; 7 Department of Epidemiology and Health Promotion, School of Public Health, Center of Postgraduate Medical Education, 01-826 Warszawa, Poland

**Keywords:** zinc, coronary artery disease, myocardial infarction, acute coronary syndrome

## Abstract

Cardiovascular diseases (CVDs) are the leading cause of death worldwide. Although zinc (Zn) was reported to have antioxidant, anti-inflammatory and protective properties in CVDs, its association with coronary artery disease (CAD) is still unclear. As methods commonly used to assess Zn levels in blood and urine do not show the full picture of the microelement supply, in this study, the nutritional status of Zn in patients with angiographically confirmed CAD was assessed using inductively coupled plasma optical emission spectrometry. We found no association between Zn and the severity of CAD evaluated with the Coronary Artery Surgery Study Score (*p* = 0.67). There were no statistically significant differences in Zn levels between patients with acute coronary syndrome and those with stable CAD (*p* = 0.937). A statically significant negative correlation was observed between Zn content and serum triglyceride concentration (*p* < 0.05). Patients with type 2 diabetes mellitus were found to have a significantly lower hair Zn content compared to non-diabetic individuals (*p* < 0.01). The role of Zn in the pathogenesis of CAD and its complications need further well-designed research as the moderation and supplementation of Zn dietary intake could be a simple intervention to reduce the CVDs risk.

## 1. Introduction

Despite great efforts to advance prophylaxis and treatment, cardiovascular diseases (CVDs) are still the leading cause of death in the world. In 2019, CVDs were responsible for 17.9 million deaths worldwide, which is 85% of deaths from myocardial infarctions or strokes. These numbers are growing, as experts predict that in 2030 CVDs-related deaths could rise to 24 million annually [1]. Despite Zinc (Zn) being one of the key microelements of the human body [2], its role in CVDs pathogenesis has not yet been firmly established. The available data generally show unfavourable levels of this metal in cardiology patients as low concentrations were found particularly in patients with CAD [3], heart failure [4,5,6] and atrial fibrillation [7]. Moreover, patients with left ventricular hypertrophy and atrial fibrillation had an inverse correlation of Zn concentration with heart muscle thickness [8]. However, data on the relationship between Zn concentration and coronary artery disease (CAD) are limited [9,10,11,12,13,14] and are mainly based on serum Zn concentration. Analytical methods commonly used to assess Zn levels in blood and urine do not show the full picture of microelement supply, as Zn content in serum is easily influenced by the time of the day, the type of meal eaten before obtaining the sample, the serum protein concentration, as well as the natural homeostasis mechanisms controlling the Zn concentration utilizing tissue storage [15]. Hair sample analysis has several advantages over serum samples. The concentration of Zn in a hair sample is about 100 times higher than in serum, and it is not as labile as serum concentration, which makes it perfect for long-term nutrition assessment [16]. In addition, the hair sample better reflects the recent excessive exposure to metals, as cations are quickly transferred from blood to tissue storage [17,18]. Hair samples are considered a good retrospective marker of microelement nutrition in the previous 6–8 weeks. As the concentration of Zn in hair is reported to reflect its content in other tissues [19,20,21], we decided to explore this approach.

This research aims to determine whether the Zn content in hair samples measured by inductively coupled plasma optical emission spectrometry (ICP-OES) correlates with the progression of CAD and acute coronary syndrome (ACS). In ICP-OES, elements are excited using heat from an argon plasma. During de-excitation, the atoms emit light with a spectrum consisting of lines specific to a particular element, allowing for the determination of the elemental composition of the sample [22]. This method has found an application in physiological samples due to its remarkable sensitivity and versatility. As obtaining a hair sample is simpler and less invasive than phlebotomy, this method could become an appropriate screening tool for patients at risk of CAD.

## 2. Materials and Methods

### 2.1. Study Population

This study is based on data obtained from 133 patients (37 women and 96 men) who underwent coronary angiography to assess the extent of CAD between 2013 and 2017 in the Department of Cardiology of Bielanski Hospital, Warsaw, Poland and agreed to participate in the study in writing. The analysis included patients whose hair was not dyed or permanently waved in segments of at least 3 cm, measuring from the scalp. Patients with an active neoplastic disease, significantly increased inflammatory markers, chronic kidney disease above stage III, taking medications or dietary supplements containing zinc, or using shampoos with an increased content of the bio-element were excluded from the study. Patients with a history of previous MI who were treated with coronary angioplasty were included in this study; however, those with thrombosis or restenosis were excluded. All patients lived in Warsaw and they had no history of occupational exposure to chemical elements. The study was carried out according to the principles of the Declaration of Helsinki and was approved by the bioethics committee of the Medical University of Warsaw.

### 2.2. Coronary Angiography

Coronary angiography is the default method for assessing stenosis in CAD [23]. The examination was performed by radial or femoral artery access. The severity of CAD was classified by three independent cardiologists using the Coronary Artery Surgery Study Score (CASSS). This scale reflects the stenosis of one, two or three arteries through a sum of points (0–3), which were assigned as follows: 1 point for stenosis of the main coronary artery (right coronary artery, circumflex branch, or anterior descending branch) exceeding 70% and 2 points for stenosis of the left main coronary artery greater than 50%. Inconclusive findings between moderate or severe stenosis were decided using fractional flow reserve measurement. The diagnosis of acute coronary syndrome (ACS) was based on criteria from the European Society of Cardiology guidelines, including the increased concentration of markers of myocardial injury with the coexistence of at least one of the following: symptoms of stenocardia, changes in the ECG suggestive of ischemia, results of imaging tests showing myocardial necrosis or coronary artery thrombus identified in coronary angiography [24].

### 2.3. Laboratory Tests

Non-permed and non-dyed hair samples, weighing between 0.2 and 0.3 g, were obtained from a few separate scalp sites at the back of the head, close to the skin. In preparation for inductively coupled plasma optical emission spectrometry (ICP-OES) the samples were washed using non-ionic detergent (Triton X-100, Sigma Aldrich Sp. z.o.o., Poznań, Poland) water solution (1:100) in an ultrasonic bath for 5 min, then rinsed sequentially with high-purity water, acetone and water and then dried to constant mass. The dry samples of hair, 0.15 g each, were dissolved in 4 mL of 65% nitric acid (Merck, Darmastadt, Germany) and 1 mL of 30% hydrogen peroxide (Merck) in a closed polypropylene tube (8 mL), then incubated in 80 °C for 30 min in a microwave station. After cooling to room temperature, the samples were diluted to a final volume of 10 mL with Milli-Q water and then analysed using an ICP-OES spectrometer (iCAP7400, Thermo Scientific, Waltham, MA, USA). The concentration of zinc in the solution, and then the total content in the hair samples, were calculated according to the previously determined standard curve.

### 2.4. Statistical Analysis

The Shapiro–Wilk test was used to assess the distribution of data. The chi-square statistic was used to identify associations between dichotomous and categorical data. The Mann–Whitney test was used to compare the values between two groups of patients. Kruskal–Wallis analysis by rank was used to determine the dependence between more than two groups. The R Spearman correlation test was used to evaluate the relationship between the variables. A two-sided *p*-value < 0.05 was regarded as statistically significant. Statistical analysis and figures were performed and created with Statistica 13 (StatSoft Inc., Tulsa, OK, USA). GraphPad Prism 5 (GraphPad Software Inc., San Diego, CA, USA, 2005) was used to create figures.

## 3. Results

### 3.1. Population Characteristics

The median age of the study population was 65 years (range: 37–95). The median BMI value was 27.7 kg/m^2^ (range: 16.9–54.1). A total of 39 (29.3%) participants had a normal body weight, 53 (39.9%) were overweight and 41 (30.8%) patients were classified as obese. A history of type 2 diabetes mellitus (t2DM) or diagnosis during the current hospitalization was found in 42 (31.6%) patients and pre-diabetes in 7 (5.3%) patients. On the basis of the lipid profile (total cholesterol—TC, LDL and HDL cholesterol, triglycerides—TG), hyperlipidaemia was assessed in 123 patients and diagnosed in over half of them despite statin treatment, i.e., in 55 (41.4%). Hypertension was present in 114 (85.7%) patients. Acute coronary syndrome (ACS) as the cause of hospitalization was diagnosed in 67 (50.4%) patients, while stable CAD was the cause in 66 (49.6%) patients. A history of myocardial infarction (MI) was noticed in 40 (30.1%) patients. Active smoking during the study was declared by 40 (30.1%) patients, and 17 (12.8%) patients had smoked in the past. Insignificant changes in the coronary arteries (CASSS 0) were found in only 22 (16.5%) patients. One-vessel coronary disease (CASSS 1) was found in 34 (25.6%) patients, two-vessel (CASSS 2) in 46 (34.6%) and three-vessel (CASSS 3) in 31 (23.3%) patients. The median Zn concentration was 166 parts per million (ppm) (range: 25–495).

### 3.2. Association between Zn Level and Severity of CAD

Table 1 presents results of measurements for the study group according to CASSS level. A significant difference in sex distribution was observed between CASSS groups. There was also a significant difference in distribution of patients with history of previous MI and cause of hospitalization.

### 3.3. Difference in Zn Level between Patients with Stable CAD and Patients with ACS

Significant differences were observed between patients with ACS and stable CAD in LDL and triglyceride (TG) levels (Table 2).

**Figure 1:**
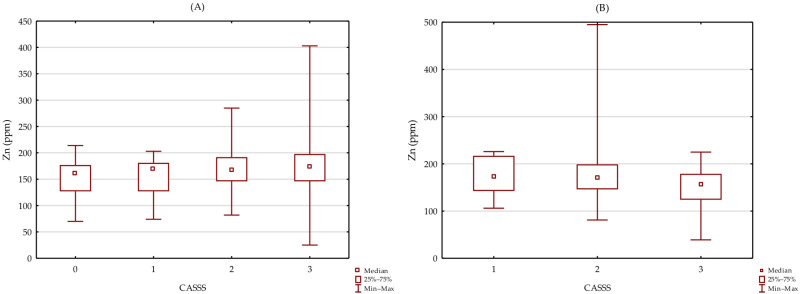
Association between Zn level and CASSS in group of patients without (**A**) and with history of MI (**B**).

A lack of significant association between Zn level and CASSS in groups of patients without (H = 2.076 *p* = 0.557; Figure 1A) and with a history of MI (H = 0.000 *p* = 1.000; Figure 1B) was observed.

**Figure 2:**
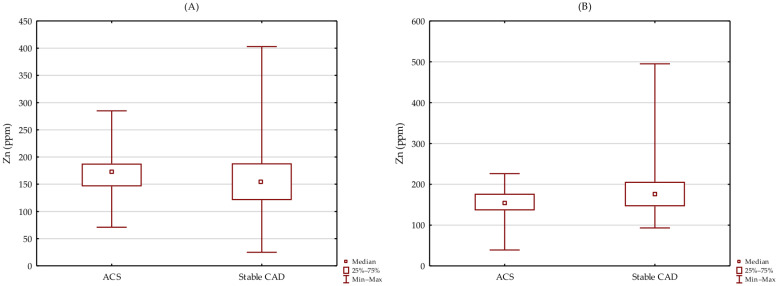
Differences in Zn level between patients with stable CAD and ACS in group of patients without (**A**) and with history of MI (**B**).

There were no significant differences in Zn level between patients with stable CAD and ACS in groups of patients without (*p* = 0.159; Figure 2A) and with a history of MI (*p* = 0.084; Figure 2B).

### 3.4. Association between Zn Level and Selected Parameters

**Figure 3:**
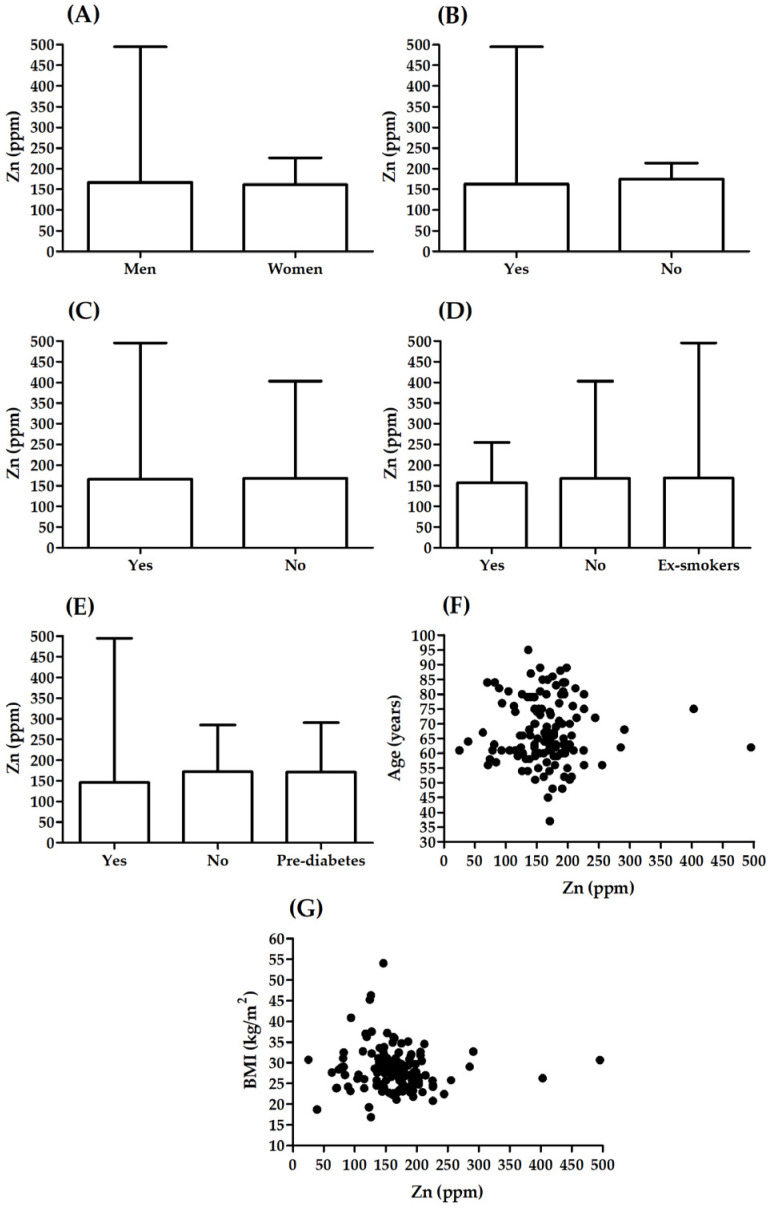
Association between Zn level and selected parameters: (**A**) sex, (**B**) hypertension, (**C**) hyperlipidaemia, (**D**) smoking status, (**E**) t2DM, (**F**) age, (**G**) BMI.

**Figure 4:**
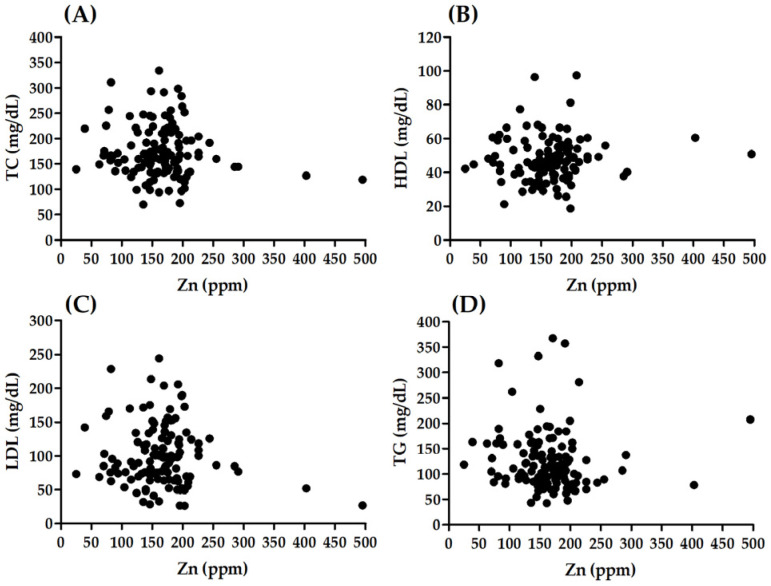
Correlation between Zn level and lipid profile: (**A**) TC, (**B**) HDL, (**C**) LDL, (**D**) TG.

There was no significant correlation between Zn and age (R = −0.005, *p* = 0.952; Figure 3F) or BMI (R = −0.15, *p* = 0.084; Figure 3G). There were no significant differences in Zn between males and females (*p* = 0.218; Figure 3A), patients with different smoking status (H 2, (N = 133) = 2073; *p* = 0.355; Figure 3D), patients with and without hypertension (*p* = 0.384; Figure 3B) and patients with or without hyperlipidaemia (*p* = 0.335; Figure 3C). Significant association was observed between Zn level and t2DM (H 2, (N = 133) = 10,952; *p* = 0.004; Figure 3E). Patients with t2DM presented significantly lower Zn values than patients without t2DM (*p* = 0.006). A lack of significant correlation was observed between Zn and TC (R = −0.070, *p* = 0.440), HDL (R = 0.091, *p* = 0.320), LDL (R = −0.020, *p* = 0.829) (Figure 4). A significant correlation was noted between Zn and TG (R = −0.193, *p* = 0.032) (Figure 4).

## 4. Discussion

This study analysed the nutritional status of Zn among patients with angiographically confirmed CAD. Intake was assessed in hair samples using ICP-OES. There was no association between the bioelement and the advancement of CAD and episodes of myocardial infarction in the analysed cohort of patients. Moreover, patients with t2DM had significantly lower Zn content in comparison to non-diabetic individuals. In this cohort, serum TG concentration was found to negatively correlate with Zn content in hair.

Zinc (Zn) is one of the key microelements of the human body [2]. Of all the transition metals, it is second only to haemoglobin-bound iron in terms of prevalence in humans [25]. It is widespread in all tissues and bodily fluids [26]. Zn deficiency has been diagnosed in 17% of the world population. This percentage increases to 35% in developing countries [27]. As Zn influences the activity of more than 300 enzymes [28], it contributes to the stabilization of many protein structures, including more than 2000 transcription factors, most notably Zn finger proteins [29]. In the human heart, more than 24 Zn transporting proteins have been identified, indicating the important role of Zn in the homeostasis of the cardiovascular system [30].

The results of previous studies indicate that Zn has a protective role in atherosclerosis, a fundamental process in CVDs. Zn and Zn-transporting proteins are important for the function of the vessel wall and its integrity. Zn is essential for the superoxide dismutase function, as it is involved in the dimerization of endothelial NO synthase and nitric oxide production. NO enables labile Zn supplies to be released from endothelial cells, which dilate the vessels and protect endothelial cells [31]. Zn deficiency allows atherosclerotic plaque to build through the aggravation of oxidative stress; destruction of NO, NF-kB and the endothelium; as well as the production of pro-inflammatory cytokines [32]. An inverse correlation was found between Zn blood concentration and the risk of CVDs [33], atherosclerosis progression [34] and CVDs complications, such as acute coronary syndrome and heart failure [35,36]. Zn also plays a key role in the immune response, as its deficiency causes a weak cellular and humoral response [37]. By regulation of the oxidation–reduction balance of cells [32], this micronutrient facilitates the integrity of the endothelial cell membrane [38] and protects it from oxidation stress [39,40]. In animal models, Zn deficiency resulted in increased levels of reactive oxygen species [41], decreased levels of glutathione [42] and superoxide dismutase 1 [43]. Supplementation reduced serum lipid peroxidation [44] and normalized inducible nitric oxide synthase activity [45]. In both animal and human models, it also suppressed the expression of pro-inflammatory cytokines regulated by NF-kB [46,47]. These effects are components of the anti-inflammatory action, which modulates the development of atherosclerotic plaques. In summary, the experimental data indicate that low levels of Zn correlate with endothelium dysfunction [47], high levels of oxidation stress and vessel wall inflammation [48]—all of them being well-established risk factors for atherosclerosis. The importance of adequate Zn intake is also supported by data suggesting a relationship between Zn deficiency and subclinical inflammation [9,49].

In this study, we did not observe a statistically significant difference in Zn levels in hair samples between subgroups of patients with different severity of CAD (CASSS 0–3). At present, the relationship between this bioelement and CAD has not yet been determined, similar to the data on CAD risk in patients and their nutritional status of Zn being equivocal. Although Islamoglu et al. suggested a direct relationship between Zn level and CAD diagnosis [10] and El-Mahdy et al. correlated lower concentrations of this microelement with a higher SYNTAX score [11], de Paula et al. suggested that Zn levels have no association with CAD course [12]. On the other hand, data from epidemiological studies show a relationship of micronutrient levels, including Zn, on the presence and progression of atherosclerosis [50,51] and CAD [9]. The ratio of Zn excretion in urine to serum concentration is well-correlated with CAD and its severity [52]. Liu et al. found that levels of serum zinc-α2-glycoprotein were decreased in patients with premature CAD [14], while Meng et al. observed that Zn levels were higher in ACS groups than in CAD groups [53]. Furthermore, their analysis revealed that serum Zn level is an independent risk factor for the development of CAD [13]. Gao et al. demonstrated an inverse correlation of Zn levels with the progression of atherosclerotic calcification in CAD [54] and the carotid intima-media thickness test as a subclinical marker of atherosclerosis [34,55]. The aforementioned articles, presenting a significant difference in Zn levels in CAD patients compared to healthy controls, are not in line with our results. This discrepancy may be due to the characteristics of the patient group whose CAD is angiographically confirmed.

In our cohort, in addition to the lack of a relationship between Zn and CAD, we did not find significant differences in the measured concentrations in patients with ACS and stable CAD. Just as the association of Zn levels with CAD is not well-established, the relationship of this micronutrient with ACS is yet to be conclusively settled. The available data show a significant decrease in Zn levels in serum and hair of patients after ACS compared to healthy controls [11,56,57]. Lower amounts of this bioelement were also observed in patients with ST-elevation myocardial infarction compared to those with non-ST-elevation myocardial infarction [11]. An inverse correlation of serum Zn concentration with the concentration of myocardial necrosis markers and clinical predictors of ACS was found [56,58]. Furthermore, the higher the concentration of Zn, the less frequently ACS was observed in a group of patients [58]. In addition to the suggested use of Zn as the prognostic marker for ACS, Lal et al. focused on the cardioprotective role of this microelement after myocardial infarction [59]. In animal models, Zn administration caused a halving of the area of the induced infarction. In addition, a reduction in the frequency of arrythmia was observed [60,61]. Taking into account the discrepancies in the results of a few randomized trials [62], some cohort research articles [33] and our results showing the lack of association of Zn in acute CAD complications, we suggest further research on this topic. A meta-analysis of prospective cohort studies by Chu et al. suggests the possibility of a correlation of higher Zn concentrations with lower CVDs risk observed in three of the five included papers. The effect of this micronutrient was more prominent in patients with t2DM who underwent angiography due to chest pain compared to the healthy population [33]. In addition, the Iowa Women’s Health Study observed an inverse correlation of dietary supplementation of Zn with CVD mortality (CAD and stroke included) in more than 30,000 postmenopausal women observed over more than ten years [62]. This result was, however, not confirmed by the recent metanalysis (49 studies with more than 300,000 participants) by Schwingshackl et al. [63]. It is worth noting that both studies used a multinutrient supplement formula with quite a small dose of Zn (≤20 mg/d), and most of the participants were healthy.

The significantly lower hair Zn content observed in patients with t2DM may indicate a correlation of this element with glucose metabolism. Experimental studies have shown that Zn plays a role in the regulation of synthesis, storage and insulin excretion from β-cells in the pancreas [64], as well as improving tissue insulin sensitivity [65,66] and regulating the activity of gluconeogenetic enzymes [67]. In diabetic patients, both hypozincaemia and hyperzincuria are frequent findings [68,69]. Supplementation with Zn reduces the risk of t2DM by up to 40% [70] and improves glucose control [71].

Our data, collected from patients with CAD treated with comparable statin doses, reveal a negative correlation between Zn content and serum TG concentration. A similar trend is observed without statistical significance for total cholesterol and LDL. These findings seem to be consistent with other studies since Zn deficiency is found to have several effects on lipid metabolism. Firstly, it decreases the TG absorption [72]. Secondly, it causes a decrease in levels of Zn-α2-glycoprotein, which in turn increases lipogenesis [73]. Supplementation with Zn decreases total cholesterol levels and increases high-density lipoprotein levels [74]. Compared to the healthy control group, lower levels of this micronutrient were found in obese patients [75]. Zn supplementation also decreases insulin resistance and inflammation marker concentrations [76].

Since almost 86% of our patients were normotensive, we were unable to prove or disprove the existence of a correlation between the nutritional status of Zn and hypertension. However, previous studies on this matter showed contradictory results [55,77,78]. Similarly, we did not find any correlation between hair Zn levels and BMI or smoking; this subject needs further in-depth research, as previous research was mainly conducted on healthy individuals [75,79].

There are a few notable limitations to this study. In particular, we did not take into consideration factors interfering with Zn levels, e.g., the potential interactions of nutritional ingredients and other microelements, which can modulate the biological effects of Zn. We omitted the differentiation of the source of Zn, which could have an influence on metabolic syndrome and the risk of CVDs [80]. We neglected the effect of drugs commonly used in CVDs, such as beta-blockers, angiotensin receptor blockers, angiotensin-converting enzyme inhibitors and diuretics. Our analysis was based on a single method of quantifying the Zn level, namely hair concentration. This omits the fact that Zn can be in different forms and its concentration changes are dynamic. The use of multiple measurement methods (hair, serum, erythrocytes, urine concentration) may result in a better understanding of the relationship between Zn and CVDs. The degree of progression of CAD was determined on the basis of the results of coronary angiography using the CASSS; perhaps using the SYNTAX or Gensini score would be more optimal.

We did not confirm an association of Zn nutritional status with CAD severity and ACS in the cohort of patients with CAD. We found a statistically significant, negative, correlation between Zn levels in hair and serum TG concentration. Furthermore, patients with t2DM had a lower Zn hair content compared to non-diabetic individuals. Evaluation of the impact of Zn on cardiovascular health requires more well-designed randomized studies to specify the advantages, dangers and contraindications for different levels of Zn dietary intake and supplementation. It is necessary to define and implement global dietary recommendations and food fortification strategies, particularly in developing countries, to achieve optimal Zn intake and reduce CVD risk.

## 5. Conclusions

In patients with angiographically confirmed CAD, there are no significant statistical differences in Zn levels between groups with different severity of CAD (CASSS 0–3). Differences in the concentration of the analysed element in hair samples were not statistically significant in patients with ACS compared to those with stable CAD. Significantly lower levels of Zn were found in the hair of patients with t2DM. A negative correlation was identified between Zn content in the hair and serum TG concentration. The role of Zn in the pathogenesis of CAD and its complications requires further well-designed research.

## Figures and Tables

**Table 1:** Association between selected parameters, including Zn level and CAD stages.

	CASSS 0	CASSS 1	CASSS 2	CASSS 3	*p*-Value
N	22	34	46	31	-
Sex (♀/♂)	10/12	12/22	6/40	9/22	0.025
Age (years)	66 (54–85)	66 (48–89)	63 (37–84)	68 (52–95)	0.455
BMI (kg/m^2^)	28.5 (21.1–46.3)	27.7 (16.9–40.9)	27.9 (22.3–54.1)	26.8 (18.7–45.3)	0.476
Cause of hospitalization (ACS/stable CAD)	5/17	22/12	22/24	18/13	0.016
t2DM (no/yes/pre-diabetes) **	17/4/1	21/13/0	29/13/4	17/12/2	---
t2DM (no/yes)	17/4	21/13	29/13	17/12	0.355
Hyperlipidaemia (no/yes) *	10/8	17/16	24/20	17/11	0.911
HDL (mg/dL) *	46.6 (28.6–66.5)	47.3 (25.7–97.4)	47.1 (21.3–77.5)	44.5 (18.6–81.3)	0.292
LDL (mg/dL) *	106.9 (26.6–171.7)	100.4 (26.2–175.3)	88.7 (26.9–244.3)	76.3 (31.8–228.3)	0.418
TG (mg/dL) *	124.8 (47.8–281.0)	104.2 (60.6–357.6)	111.6 (42.6–367.8)	110.3 (43.4–332.4)	0.545
TC (mg/dL) *	178.2 (73.3–255.9)	166.9 (101.7–256.7)	159.9 (94.1–334.1)	154.5 (70.0–310.8)	0.316
Hypertension (no/yes)	5/17	6/28	4/42	4/27	0.421
History of MI (no/yes)	22/0	26/8	27/19	18/13	0.002
Smoking (no/yes/ex-smokers) **	16/5/1	22/10/2	19/17/10	19/8/4	---
Smoking (no/yes)	16/5	22/10	19/17	19/8	0.254
Zn (ppm)	161 (70–214)	169 (74–226)	169 (81–495)	166 (25–403)	0.670

*—assessed in 123 patients; **—three subgroups of patients due to the low number of patients in the selected subgroup; data for three and two groups are presented separately for statistical purposes.

**Table 2:** Differences in selected parameters between patients with ACS and stable CAD.

	ACS	Stable CAD	*p*-Value
N	67	66	-
Sex (♀/♂)	16/51	21/45	0.307
Age (years)	66 (37–95)	63 (51–85)	0.345
BMI (kg/m^2^)	27.7 (16.9–45.3)	27.6 (19.3–54.1)	0.791
t2DM (no/yes/pre-diabetes) **	44/19/4	40/23/3	---
t2DM (no/yes)	44/19	40/23	0.449
Hyperlipidaemia (no/yes) *	35/26	33/29	0.643
HDL (mg/dL) *	46.6 (25.7–81.3)	46.0 (18.6–97.4)	0.279
LDL (mg/dL) *	101.5 (26.6–244.3)	78.6 (26.2–204.0)	0.008
TG (mg/dL) *	103.3 (42.6–367.8)	124.7 (66.6–357.6)	0.008
TC (mg/dL) *	168.3 (70.0–334.1)	159.6 (96.4–291.1)	0.142
Hypertension (no/yes)	8/59	11/55	0.436
History of MI (no/yes)	49/18	44/22	0.416
Smoking (no/yes/ex-smokers) **	43/22/2	33/18/15	---
Smoking (no/yes)	43/22	33/18	0.871
Zn (ppm)	166.0 (39.0–285.0)	166.5 (25.0–495.0)	0.937

*—assessed in 123 patients; **—three subgroups of patients due to the low number of patients in the selected subgroup; data for three and two groups are presented separately for statistical purposes.

## Data Availability

Data can be provided by the corresponding author upon reasonable request.
